# Special Issue: Antifungal Agents Recently Approved or under Development

**DOI:** 10.3390/jof7030239

**Published:** 2021-03-23

**Authors:** Ana Espinel-Ingroff, Eric Dannaoui

**Affiliations:** 1 Virginia Commonwealth University Medical Center, Richmond, VA 23219, USA; 2 Unité de Parasitologie-Mycologie, Service de Microbiologie, Hôpital Européen Georges Pompidou, F-75015 Paris, France; 3 Faculté de Médecine, Université de Paris, F-75006 Paris, France

Many thanks to all contributors to the Special Issue on “Antifungal Agents Recently Approved or Under Development (Current Knowledge and Future Perspectives)”. The aim was to highlight and summarize current data for eight novel agents, including isavuconazole, developed for treating invasive fungal infections: (i) the fungal Gwt1 protein inhibitor manogepix (fosmanogepix (APX001; E1210), (ii, iii) the two glucan synthase inhibitors, rezafungin (CD101) and ibrexafungerp (SCY-078), (iv) the orotomide, olorofim (F90131), (v) a new triazole, PC945, as well as (vi) encochleated amphotericin B, (vii) isavuconazole, and (viii) the chitin synthase inhibitor, nikkomycin. The reported characteristics of each agent were reviewed as follows: (i) molecular mechanisms of action and resistance, (ii) in vitro activity, (iii) pharmacokinetics/phamacodynamic (PK/PD) data, (iv), safety and clinical efficacy from clinical trials as summarized below.

Both intravenous (IV) and oral formulations of manogepix (fosmanogepix) are being developed for the convenient continuation of care outside the hospital with a single dose (phase 2 clinical trials and orphan drug approval). A favorable drug–drug interaction profile and a wide tissue distribution make it an ideal agent for empiric or front-line therapy. Extensive MIC data for both yeasts (*Candida auris* included) and molds were summarized [1].

As with the established echinocandins, rezafungin MICs are low including resistant/mutant *Candida*, *C. auris* and *Aspergillus* isolates and the presence of *FKS* mutants at a similar frequency [2]. It has no activity against the Mucorales, *Fusarium* spp. and *Ajellomycetaceae*; it also shows the lowest frequency of paradoxical effect and trailing [2,3]. It has better tissue penetration, prolonged pharmacokinetics/phamacodynamic (PK/PD), pharmacometrics, and a better safety profile than established echinocandins. Because of that, rezafungin could be administered IV once weekly (prophylaxis development) [4]. In a similar manner, ibrexafungerp, a new triterpenoid that inhibits beta-1-3-D-glucan synthesis, has low MICs for resistant isolates of *C. auris*, *Candida* and *Aspergillus* mutants. The in vivo activity of both new glucan synthase inhibitors was excellent in candidiasis clinical trials (especially for acute vulvovaginal candidiasis included) and aspergillosis [4,5]. 

Olorofim is an investigational agent currently in clinical studies for the treatment of refractory/resistant invasive mold infections and thermally dimorphic fungi; however, this agent has no activity against yeasts (*Candida* spp. and *Cryptococcus* spp.) and Mucorales [6]. 

PC945 is a novel triazole, designed for administration via pulmonary inhalation, leading to high local concentrations; however, it has low systemic exposure. It is a promising agent for the treatment of pulmonary *Aspergillus* infections in patients with chronic lung diseases [7]. On the other hand, the established triazole, isavuconazole, has had clinical use authorization since 2015. It is as effective as voriconazole for the treatment of invasive aspergillosis and to liposomal amphotericin B for mucormycosis, but not for candidemia [8]. It has a broad spectrum of in vitro activity and predictable pharmacokinetics. The mechanisms of action/resistance were also explored [9].

One of the main advantages of encochleated amphotericin B is its reduced toxicity and potential oral administration [10]. Nikkomycin inhibits chitin synthesis and has in vitro activity against fungal species that usually require long term therapy, such as those caused by *Coccidioides immitis*; it has shown some activity in dog infections and synergistic activity in animals against *Candida* and *Aspergillus* infections. The value of this agent should be demonstrated in clinical trials [11]. 

In conclusion, these reviews provide a wealth of information on what is currently known about the various antifungal agents under development and isavuconazole. It is expected that these agents could help in dealing with the increased incidence of severe fungal infections and the emerging antifungal resistance among *Candida* and *Aspegillus.* Again, many thanks to the contributors for their time and expertise despite their busy schedules due to the COVID-19 pandemic.
